# Fecal microbial gene transfer contributes to the high-grain diet-induced augmentation of aminoglycoside resistance in dairy cattle

**DOI:** 10.1128/msystems.00810-23

**Published:** 2023-12-12

**Authors:** Tao Zhang, Yingyu Mu, Yunlong Gao, Yijun Tang, Shengyong Mao, Jinxin Liu

**Affiliations:** 1 Ruminant Nutrition and Feed Engineering Technology Research Center, College of Animal Science and Technology, Nanjing Agricultural University, Nanjing, China; 2 Laboratory of Gastrointestinal Microbiology, Jiangsu Key Laboratory of Gastrointestinal Nutrition and Animal Health, National Center for International Research on Animal Gut Nutrition, College of Animal Science and Technology, Nanjing Agricultural University, Nanjing, China; The University of Maine, Orono, Maine, USA

**Keywords:** high-grain diet, microbiome, gut resistome, microbial lateral gene transfer, dairy cattle

## Abstract

A high-grain (HG) diet can rapidly lower the rumen pH and thus modify the gastrointestinal microbiome in dairy cattle. Although the prevalence of antibiotic resistance is strongly linked with the gut microbiome, the influences of HG diet on animals’ gut resistome remain largely unexplored. Here, we examined the impact and mechanism of an HG diet on the fecal resistome in dairy cattle by metagenomically characterizing the gut microbiome. Eight lactating Holstein cattle were randomly allocated into two groups and fed either a conventional (CON) or HG diet for 3 weeks. The fecal microbiome and resistome were significantly altered in dairy cattle from HG, demonstrating an adaptive response that peaks at day 14 after the dietary transition. Importantly, we determined that feeding an HG diet specifically elevated the prevalence of resistance to aminoglycosides (0.11 vs 0.24 RPKG, *P* < 0.05). This diet-induced resistance increase is interrelated with the disproportional propagation of microbes in Lachnospiraceae, indicating a potential reservoir of aminoglycosides resistance. We further showed that the prevalence of acquired resistance genes was also modified by introducing a different diet, likely due to the augmented frequency of lateral gene transfer (LGT) in microbes (CON vs HG: 254 vs 287 taxa) such as Lachnospiraceae. Consequently, we present that diet transition is associated with fecal resistome modification in dairy cattle and an HG diet specifically enriched aminoglycosides resistance that is likely by stimulating microbial LGT.

**IMPORTANCE:**

The increasing prevalence of antimicrobial resistance is one of the most severe threats to public health, and developing novel mitigation strategies deserves our top priority. High-grain (HG) diet is commonly applied in dairy cattle to enhance animals’ performance to produce more high-quality milk. We present that despite such benefits, the application of an HG diet is correlated with an elevated prevalence of resistance to aminoglycosides, and this is a combined effect of the expansion of antibiotic-resistant bacteria and increased frequency of lateral gene transfer in the fecal microbiome of dairy cattle. Our results provided new knowledge in a typically ignored area by showing an unexpected enrichment of antibiotic resistance under an HG diet. Importantly, our findings laid the foundation for designing potential dietary intervention strategies to lower the prevalence of antibiotic resistance in dairy production.

## INTRODUCTION

The increasing prevalence of antibiotic-resistant bacteria and antibiotic-resistance genes (ARGs) has become a severe public health concern, as they can compromise the treatment of clinical bacterial infections in humans and animals (1–4). The gut microbiome of animals has been identified as a significant reservoir of ARGs (5–7) and from which antibiotic resistance is found widely shared with the human gut (8, 9). Dairy cattle are the primary provider of meat and dairy products and are closely related to food safety (10, 11). Therefore, studies examining the gut resistome in dairy cattle are vital to address the public health issues related to sustainable dairy farming and mitigations of antibiotic resistance.

Previous studies indicated the potential influence of dietary interventions on gut resistome (12, 13), including an increased diversity of gut resistome induced by a high-protein diet in humans (13), and a fiber-rich diet contributes to lower the prevalence of gut resistance genes (RGs) in companion animals (12). Although efforts were made to characterize the baseline-level prevalence of antibiotic resistance in dairy cattle (14–16), studies examining the interaction between diet and gut resistance for potential dietary interventions remain unavailable. In intensive farming, a high-grain (HG) diet is commonly used to improve economic efficiency, but it increases the risk of intestinal microecological disorders and metabolic diseases in animals (17–19). Studies have indicated that the diet is linked with the development of gut resistome in livestock, and an increased prevalence of specific ARGs, including resistome to macrolide and streptomycin, was found in the rumen of dairy cattle when transitioned from forage to an HG diet (7, 20). However, a mechanistic understanding of the influences of different types of diets on the fecal resistome in dairy cattle is currently lacking. Given that livestock feces play a direct and significant role in contaminating the environment with microbial hazards, it is vital to understand which diet works better in controlling the prevalence of fecal antimicrobial resistance.

We aim to explore the response of gut resistome to the dietary transition from forage to HG diets in dairy cattle and hypothesize that the proliferation of antibiotic-resistant bacteria and microbial lateral gene transfer (LGT) jointly contribute to the modifications in the fecal resistome. We argue in the present study that dietary intervention is a promising strategy for controlling antibiotic resistance in food-producing animals.

## RESULTS

### Shotgun metagenomic sequencing and experimental grouping

Approximately 306 million high-quality sequencing reads with an average of 10.2 million sequences per sample (*N* = 30) were obtained with metagenomic sequencing following quality control and host genome filtration (Fig. S1a and b). We tailored our analyses specifically on bacteria and archaea as these are often the microbial hosts of RGs. To eliminate artificial grouping bias, we examined the microbial diversity of rectal feces from dairy cattle before dietary transition (i.e., forage to HG diet). There was no statistical difference observed either in alpha diversity (Fig. S2a to c; Simpson index, *P* > 0.05) or microbial composition (NMDS analysis, Adonis, *P* > 0.05) between the conventional (CON) and HG groups, indicating a successful random allocation of animals.

### High-grain diet dramatically influences the fecal microbiome in dairy cattle

**Fig 1:**
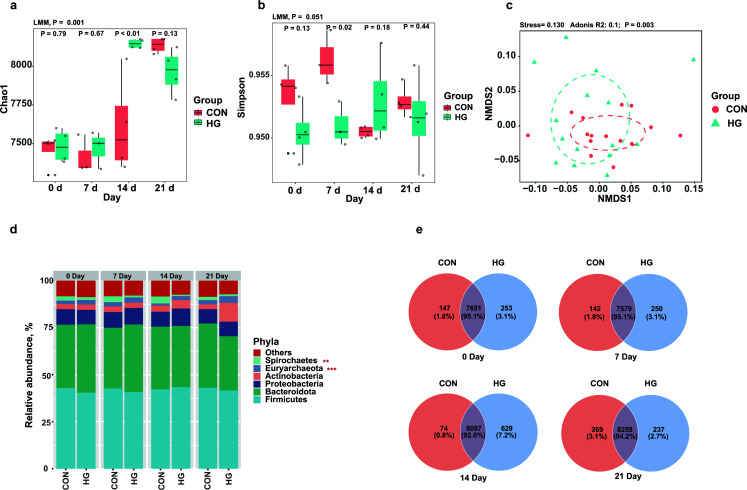
The fecal microbiome’s composition were compared between dairy cattle fed a conventional (CON) diet and a high-grain (HG) diet. The assessment of fecal microbial richness (**a**) and alpha-diversity (**b**) revealed a more diversified microbiome in the HG groups (LMM, *P* < 0.05). (**c**) NMDS analysis displayed a distinct separation between the CON and HG groups based on the Bray–Curtis metric (Adonis, *P* = 0.003). (**d**) The relative abundance of dominant phyla (≥1% in all samples) in the fecal microbiome of dairy cattle. (**e**) Venn diagram analysis showed that the HG group possessed a greater number of unique microbial taxa on day 14 after the dietary transition.

Introducing the HG diet significantly increased the richness of the fecal microbiome (Fig. 1a, *P* < 0.05), with the most pronounced effect observed on the 14th day and an unexpected increase from the control on day 21 (Fig. 1a). The alpha diversity as measured by the Simpson index indicated a similar trend but a slight discrepancy between two groups (Fig. 1b, *P* = 0.051). As expected, our ordination analysis indicated that the microbial structure was distinct in animals fed with an HG and forage diet (Fig. 1c, Adnois, *P* = 0.003). Moreover, analysis with Bray–Curtis dissimilarity, a measure of bacterial community stability, demonstrated that the most significant variation occurred at day 14 (Fig. S3a, *P* < 0.001) following the dietary transition. Overall, we observed a dynamic change of in the fecal microbiome to an HG diet in dairy cattle, with the most prominent variation manifested on day 14.

We next taxonomically profiled the fecal microbiome, and Firmicutes (42.2%) and Bacterodietes (33.5%) were found to be the primarily dominant phyla across samples (Fig. 1e). Using differential abundance analysis with ANCOM-BC, we observed a substantial enrichment of Euryarchaeota (*P* < 0.001, Fig. 1d) in the HG group, whereas the abundance of Spirochaetes was notably diminished (*P* = 0.035, Fig. 1d). At the family level, taxa often associated with polysaccharide degradation, namely, Lachnospiraceae (13.3%) and Oscillospiraceae (12.0%) were dominant in this group, while the abundance of Methanobacteriaceae, known for producing methane, was significantly enriched in the HG group (*P* = 0.001, Fig. S3b). Interestingly, the relative abundance of Lachbospiraceae gradually increased after introducing an HG diet and peaked at day 14 (Fig. S3c). Our Species-level analysis further revealed a higher number of unique microbial taxa in the HG group on day 14 (7.2%, Fig. 1e), and many of these belong to bacterial families Streptomycetaceae, Muribaculaceae, and Lachnospiraceae (Fig. S3d).

### Influences of dietary transition on dairy cattle fecal resistome

**Fig 2:**
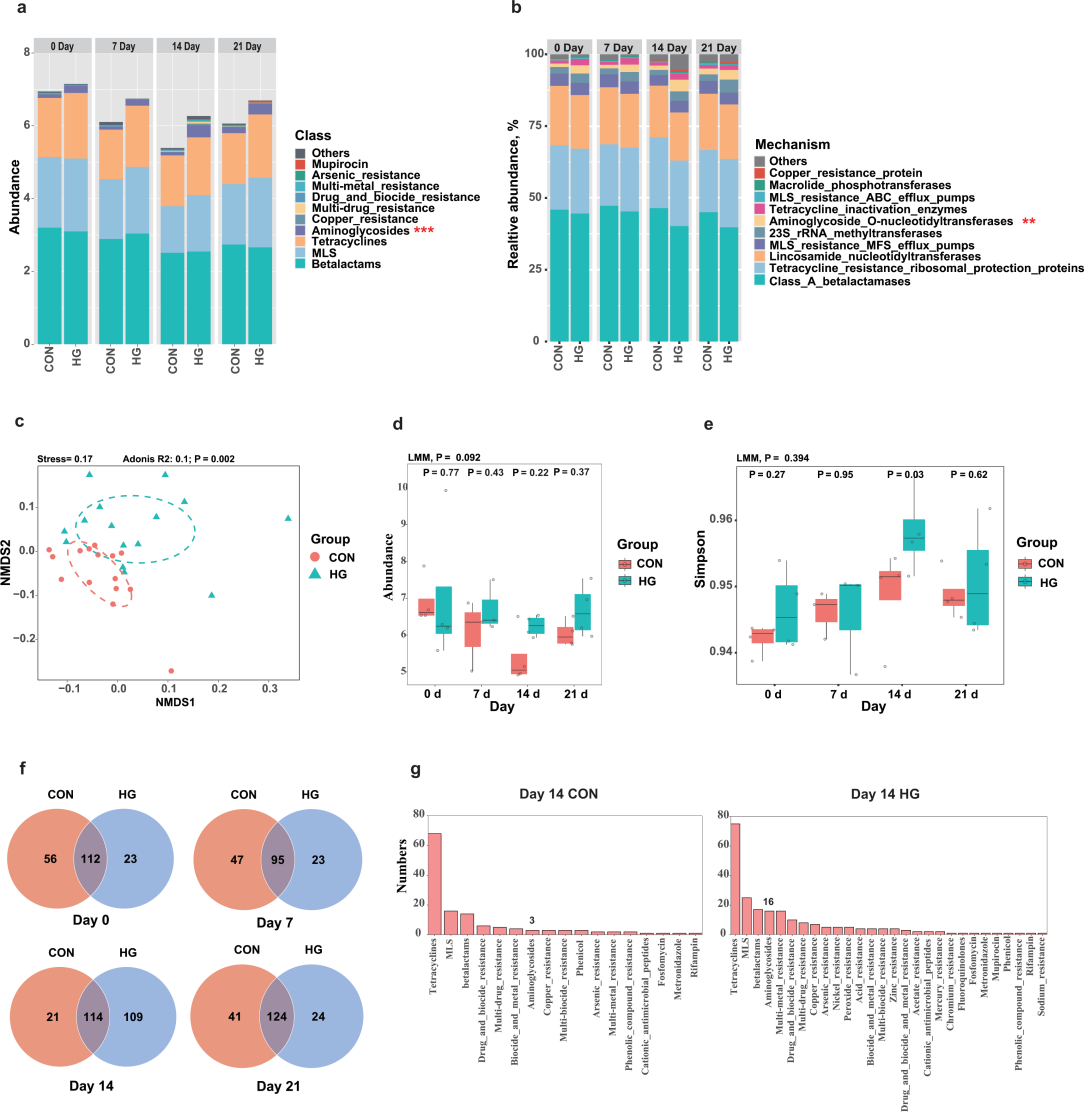
Analysis of fecal resistome’s composition and mechanism in dairy cattle between the conventional (CON) and high-grain (HG) groups. (**a**) Fecal resistome composition was summarized at the antimicrobial-class level (top 10) that supports the aminoglycosides resistance enrichment in the HG group (LMM, ***P < 0.01). (**b**) The analysis of antimicrobial resistance mechanisms (≥ 1% in all samples) in the fecal microbiome demonstrated an elevated presence of aminoglycoside_O-nucleotidyltransferase in the HG group (LMM, **P < 0.05). (**c**) The structure of fecal resistome was distinct between the CON and HG groups based on NMDS analysis (Bray–Curtis metric, Adonis, *P* = 0.002). (**d**) The total abundance of fecal resistance genes was slightly increased following the intake of an HG diet (LMM, *P* = 0.092). (**e**) The fecal resistome exhibits similar alpha diversity between the CON and HG groups (LMM, *P* = 0.394). (**f and g**) Venn’s analysis of resistance gene types indicated that the HG group possessed a greater number of resistance genes on day 14, particularly showing an increase in the number of aminoglycosides resistance genes (CON vs HG: 3 vs 16 RGs).

With MGEARes 2.0, our fecal resistome analysis identified 320 unique RGs, collectively representing 30 classes of antimicrobials and 68 resistance mechanisms. Overall, the most prevalent resistance phenotype was drug resistance (98.8%, with 190 unique RGs), followed by metal (0.7%, with 61 unique RGs), multi-compound (0.3%, with 40 unique RGs), and biocides resistance (0.2%, with 29 unique RGs) (Fig. S4a). β-lactams resistance represents a significant portion of dairy cattle fecal resistome (44.3% on average), followed by resistance to Macrolide-lincosamide-streptogramin (MLS, 26.7%), tetracyclines (24.5%), and aminoglycosides (2.8%) (Fig. 2a). Among these, aminoglycoside RGs were notably enriched in animals fed with an HG diet (LMM, *P* < 0.01). Consistent with this, our resistome analysis at the resistance mechanisms level found that classs_A_β-lactamases (44.2%), tetracycline_resistance_ribosomal_protection_proteins (22.7%), and Lincosamide_nucleotidyltransferases (18.9%) were the most prevalent groups, with aminoglycoside_O-nucleotidyltransferase (LMM, *P* < 0.001) being the significantly more enriched resistance mechanism in the HG diet group. ARGs, including *cfx*, *lnuC*, *tetQ*, *tetW*, *tet40*, *mefA*, *tetO*, *ermF*, *acI*, and *tet32,* were the most dominant genes in this study (Fig. S4b). Our LMM analysis revealed that *tet40* (*P* < 0.048) and *ant9* (*P* < 0.036) were more prevalent in the HG group compared to the CON.

Overall, diet is a significant influencer in separating the fecal resistome structure from animals treated with an HG or CON diet (NMDS, Adonis, *P* = 0.002, Fig. 2c). The normalized abundance of RGs was found to be higher in the HG group (6.71 ± 0.30 RPKG) (mean ± SD) than in animals from the control (6.12 ± 0.22 RPKG) (LMM, *P* = 0.092, Fig. 2d). Although the Simpson’s index did not show a significant difference between the two groups (LMM, *P* = 0.394, Fig. 2e), we observed the greatest variation on day 14 (*P* = 0.03, Fig. 2e). In addition, we found that a greater number of different RGs were present in the HG group (*N* = 109) compared to the CON group (*N* = 21) on day 14 (Fig. 2f). Specifically, the HG diet significantly enriched the number of ARGs related to aminoglycosides (CON vs HG: 3 vs 16 RGs, Fig. 2g).

### The inferred microbial hosts of RGs in dairy cattle

**Fig 3:**
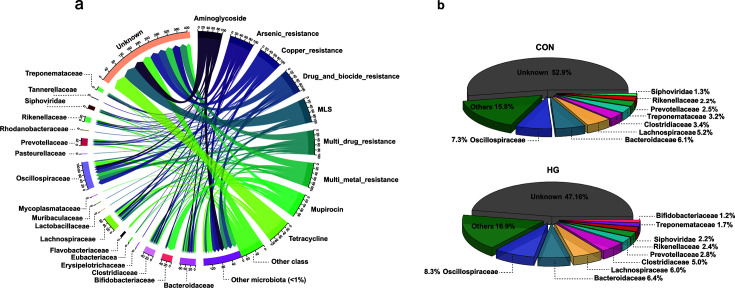
The inferred microbial host distribution of fecal resistome in dairy cattle. (**a**) The characterized RGs, categorized by classes of antibiotics, to the corresponding predicted microbial hosts at the family level. The Y-axis represents the proportion of RGs carried by different microbial families. Only families carrying a proportion of ARGs greater than 1% of the total ARGs carried by all families are displayed. (**b**) The proportion of microbial hosts (≥1% in all samples) in the conventional and high-grain groups.

Resistance genes are often disproportionally distributed across bacterial taxa; hence, the observed effect of dietary transition on the fecal resistome is likely a result of modifications in the microbiome. Our RGs origins analysis revealed that 52.1% of microbial hosts of RGs were successfully identified with a known taxon, and primarily within bacterial families Oscillospiraceae (8.8%), Bacteroidaceae (7.4%), and Lachnospiraceae (5.5%) (Fig. 3a). Given the differing prevalence of resistance to aminoglycosides in the two groups, we specifically characterized the microbial hosts of these genes. We found that they were predominantly carried by Oscillospiraceae (7.28% vs 8.26%), Bacteroidaceae (6.13% vs 6.43%), and Lachnospiraceae (5.2% vs 5.99%) in the CON and HG groups, respectively (Fig. 3b). Consequently, the gradual increase in prevalence of Lachnospiraceae likely contributes to the enrichment of resistance to aminoglycosides.

### The prevalence of transferrable RGs is elevated in animals supplied with an HG diet

**Fig 4:**
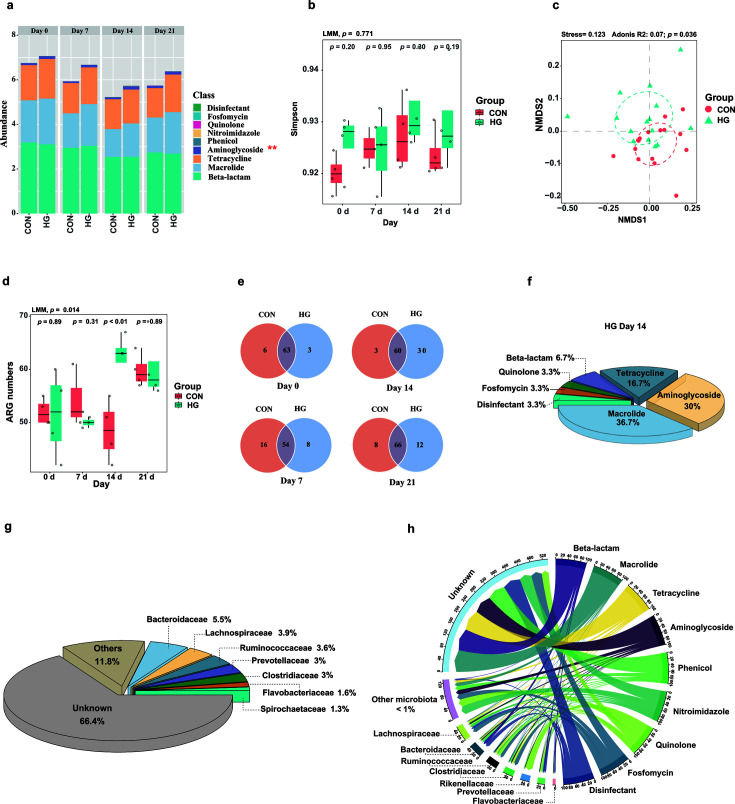
The characterization of fecal mobilome in dairy cattle. (**a**) A stacked bar plot depicting the absolute abundance of acquired resistance genes showed that the aminoglycosides resistance genes were more enriched in the high-grain (HG) group (LMM, **P < 0.05). (**b**) Boxplot demonstrating the alpha diversity of acquired resistance genes. (**c**) NMDS analysis reveals the distinct distribution of acquired resistance genes between the conventional (CON) and HG groups (Adnois, *P* = 0.036). (**d**) Boxplot showing a significant difference in acquired ARG numbers between the CON and HG groups (*P* = 0.014). (**e**) The Venn diagram reveals a higher count of unique acquired resistance genes in the HG group on day 14, and the pie plot (**f**) shows their predominant distribution. (**g**) Pie plot demonstrating the composition of the acquired ARG bacterial host in all samples (≥ 1% ) (**h**) Chord diagram connecting acquired resistance genes and inferred microbial hosts at the family level. Bacterial families and corresponding genes that contributed to less than 1% of overall resistance were filtered out for better visualization.

The observed modifications in dairy cattle fecal resistome cannot be fully explained by clonal expansion of particular taxa, raising the possibility of differed capability in horizontal gene transfer. Therefore, we specially examined the profile of the acquired RGs with the Resfinder (v 4.1) in dairy cattle fecal microbiome. A total of 115 different transferable ARGs, which contributed to resistance against nine different antibiotics, were identified. These genes collectively confer resistance to β-lactam (46.2%) (mean), macrolide (27.1%), tetracycline (24.8%), and aminoglycosides (1.6%). Consistent with the aforementioned findings concerning aminoglycosides, resistance to this class of antibiotic with transfer potentials exhibited a significantly higher prevalence in the HG group than in controls (LMM, *P* = 0.036, Fig. 4a).

Although the analysis of the Simpson index (LMM, *P* = 0.771, Fig. 4b) indicated no statistically significant impact from an HG diet on the alpha diversity, the resistome was distinct in these two groups, as revealed by the NMDS analysis (Bray–Curtis, Adnois, *P* = 0.036, Fig. 4c). The number of observed RGs with transferrable potential was statistically different in the two groups (median of 53 vs 56 for CON and HG, respectively). A gradual increase in the number of unique acquired RGs in the HG group (peaking on day 14) after introducing an HG diet was also documented in our cohort (Fig. 4d and e). Among genes from the HG group, 30% were predicted to confer resistance to aminoglycosides (Fig. 4f). Our microbial host analysis indicated that Bacteroidaceae (5.5%) was the dominant family carrying acquired RGs, followed by Lachnospiraceae (3.9%), Ruminococcaceae (3.6%), and Prevotellaceae (3.0%) (Fig. 4g and h).

### The HG diet increases the frequency of LGT in dairy cattle fecal microbiome

**Fig 5:**
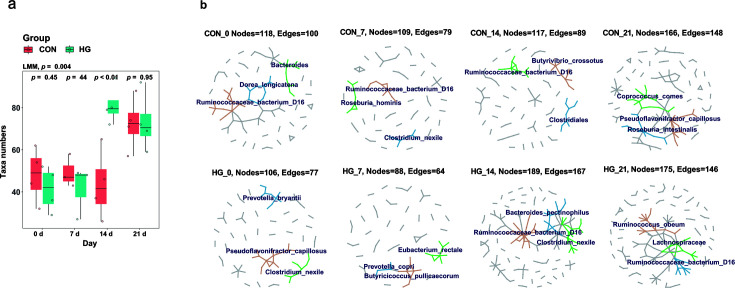
Analysis of fecal microbial LGT events in dairy cattle. (**a**) Boxplot demonstrating the microbial taxa involved in LGT across different sampling points. (**b**) Network analysis predicted LGT events in the fecal microbiome between the conventional and high-grain groups. At each time point, three representative microbial clusters and the corresponding nodes (hub taxa) were colored to depict a gradually modified network; hub taxa are labeled from top to bottom, indicating a decreased occurrence.

To further evaluate the impact of diet on gene transfer in dairy cattle, we examined the occurrence of LGT in the fecal microbiome. Overall, 353 microbial taxa were found to be involved in LGT events, with a higher number in the HG group (*N* = 287 taxa) compared to the CON group (*N* = 254 taxa). Interestingly, after 14 days of dietary transition, we observed that a significantly higher number of microbial taxa was involved in LGT in the HG group (LMM, *P* = 0.004, Fig. 5a). Our network modeling analysis identified 14 key taxa that were predicted with the most occurrence of LGT across all samples, representing microbes belonging to Lachnospiraceae, Ruminococcaceae, Clostridiaceae, Prevotellaceae, Eubacteriaceae, and Bacteroidaceae. Furthermore, the gene transfer network in the HG group was noticeably more complex than that in the CON group on day 14 (Edges: 167 vs 89, Fig. 5b). Consequently, we suggest that the high prevalence of acquired RGs was reflected in the LGT in fecal microbiota after being fed an HG diet for 14 days.

## DISCUSSION

Previous studies have demonstrated the rumen microbiome is subjected to change following diet transition from forage to HG (21). This modification will likely influence the prevalence and distribution of antimicrobial resistance in the rumen of dairy cattle (22). This is at least particularly because ARGs often disproportionally distribute across microbial taxa, and the capability of microbial horizontal gene transfer may also vary under different diets (23). Despite that, a mechanistic understanding of dietary transition’s influences on dairy cattle’s gastrointestinal resistome is still lacking; insights on this will help design dietary interventions in lowering the prevalence of antibiotic resistance in dairy production.

We examined the dietary transition (i.e., forage to HG diet) on dairy cattle’s fecal microbiome and resistome within 3 weeks. We found that the microbial community and fecal resistome were modified, with the greatest variation observed on day 14. This is consistent with previous studies that indicated that when experiencing feed transitions, the gut microbiota was gradually altered, indicating an adaptive response to accommodate the new diet (24, 25). Obviously, the fecal microbiota responds rapidly to HG diets by promoting the proliferation of Methanobacteriaceae (Euryarchaeota), a prevalent family of methane-producing bacteria capable of consuming easily digested carbohydrates (26). In addition, the elevation of easily fermentable carbohydrates in the diet stimulates a rise in the population of polysaccharide-degrading bacteria within the cattle gut, including Muribaculaceae (27) and Lachnospiraceae (28). This find highlights their unique role in digesting complex carbohydrates. Overall, we observed an adaptive response of the fecal microbiome to the dietary transition during the first 2 weeks, with microbial communities slightly recovered afterward.

Comprehensively, we observed that fecal microbes predominantly exhibited resistance to β-lactams, MLS, and tetracycline, aligning with their widespread application in commercial farms in China (29–31). This observation further suggests the enduring presence of these resistance phenotypes in the dairy population. The regulatory role of feed on gut resistome has been discussed previously (12, 32, 33), and Mu et al. recently observed an elevated prevalence of antibiotic resistance in the rumen of dairy cattle under an HG diet compared to CON feed (20). The present study expanded the analysis to fecal resistome, which is more likely to contaminate the downstream environment than rumen samples (34, 35), and determined that feeding an HG diet specifically elevated the prevalence of resistance to aminoglycosides, particularly by the end of 2 weeks.

The gut and environmental resistome have been suggested to be strongly linked with the microbial community composition (36–39). Our microbial origin analysis indicated that bacterial families Oscillospiraceae, Bacteroidaceae, and Lachnospiraceae are frequently characterized as the bacterial hosts of RGs. This observation is consistent with a previous study, where the authors identified Lachnospiraceae as one of the most prevalent bacterial hosts of RGs in the rumen of dairy cows (40). Consequently, we infer that the HG diet-induced elevation of resistance can be at least partially explained by the disproportional proliferation of Lachnospiraceae in animals from these two groups.

The mobility of ARGs poses another threat to the escalating prevalence of antibiotic resistance (41–44) on top of the clonal expansion of antibiotic-resistant bacteria. Our mobilome analysis further observed that feeding an HG diet significantly elevated the abundance of acquired RGs to aminoglycosides in dairy cattle. The microbial origin analysis indicated that these transferrable RGs were predominantly found in Bacteroidacea, Lachnospiraceae, and Ruminococcaceae. Coincidentally, Lachnospiraceae was the bacterial family that is most frequently characterized to harbor aminoglycoside-associated acquired RGs. These results suggest that the introduction of an HG diet likely promoted the transfer of RGs among microbes, such as Lachnospiraceae, resulting in an enrichment of ARGs, particularly to aminoglycosides. This aligns with our LGT-focused observation that more LGT events were found in the HG group compared to the controls, and Lachnospiraceae emerged as a key family for gene transfer.

## MATERIALS AND METHODS

All the procedures in the current experiment were conducted according to the Animal Protection Law based on the Guide for the Care and Use of Laboratory Animals approved by the Ethics Committee of Nanjing Agricultural University under protocol no. SYXK-2017–0027.

### Animal experimental design

Eight healthy mid-lactating Holstein cows (579.3 ± 53.3 kg) were randomly selected from a commercial dairy farm and were housed in individual tie-stalls for 4 weeks. To effectively monitor significant shifts in gut microbiota and mitigate prolonged exposure of dairy cows to subacute ruminal acidosis, we implemented a relatively brief intervention period of 3 weeks. All cattle were fed a forage-based diet for 1 week before the experiment. After the diet adaption, four cattle were further randomly chosen into the CON group and kept feeding the foraged-based diet. The other four cattle were distributed to feed on an HG diet with forage/concentrate ratios of 4:6 on a dry matter basis (Table S1). Cattle were fed twice during our experimental period at 07꞉00 and 19꞉00 (5% to 10% orts on an as-fed basis) daily.

### Rectal feces collection

Before the dietary adjustment (i.e., day 0), the rectal feces were collected from all cattle using long-arm sterile gloves. During the trial period, rectal feces were collected on the last day (days 7, 14, and 21) each week. All fecal samples were divided into freezer-storage tubes under sterile conditions and stored in liquid nitrogen until microbial DNA extraction.

### DNA extraction and metagenomic sequencing

Total DNA was extracted from all rectal feces (~200 mg per sample) using a microbead oscillator with repeated bead beating (Biospec Product, Bartlesville, OK, USA) (45). The integrity of the extracted DNA was subjected to rigorous scrutiny based on 1% gel agarose-electrophoresis and Nanodrop ND-1000 (Thermo Scientific, Wilmington, USA) dual assay. According to the specification of the TruSeq DNA PCR-Free Library Preparation Kit (Illumina, San Diego, CA, USA), high-quality DNA from each sample was used to construct a metagenomic library and sequenced on an Illumina NovaSeq 6000 platform.

### Sequences quality control and metagenome assembly

In total, 30 fecal samples (one sample was removed from each group on day 7 because of low sequencing quality) from eight dairy cattle at 4-time points (day 0, day 7, day 14, day 21) were used to proceed with metagenomic sequencing. Bowtie2 (46) removed reads aligning to the bovine genome (v ARS-UCD 1.2) to eliminate potential host contamination. The subsequent cleaned data further went through quality control with Trimmomatic (v 0.35, LEADING:3 TRAILING:3 SLIDING WINDOW: 4:15) (47) and duplicated sequences were removed by using FastUniq (v 1.1). BBMap (repair.sh, v 38.72) was used to repair and sort the resulting sequences. BBmap was also used to assess the trimmed sequencing depth and read length, and fastqc (v 0.11.5) was employed to examine the sequence quality with default settings. Bracken and Kraken2 (48) were used to taxonomically profile the metagenomes with the default database (i.e., Minikreaken2_v1_8 GB, which includes bacteria, archaea, and viral). MEGAHIT (v 1.2.9) (49) was used to assemble metagenomes into contigs per sample with default parameters.

### Resistome analysis

Sequencing reads were aligned to MEGARes 2.0 (50) to characterize the resistome composition (including antibiotics, heavy metals, biocides, and multi-compounds). Briefly, BWA (51) was used to map the merged reads to the database with default settings, and the SAM file was then analyzed through ResistomeAnalyzer (v 1.0) by setting the threshold to 80% identify (https://github.com/cdeanj/resistomeanalyzer). Putative RGs were analyzed at five levels (i.e., Type, Class, Mechanism, Group, and MEGID). Normalization was performed for downstream comparisons by calculating the number of RGs per estimated genome using a custom script adapted from Li et al. (52). Average genome size was obtained by analyzing sort sequences with MicrobeCensus (v 2.15) with an adjusted parameter (-n 100000000) (53). The abundance of RGs, as quantified by resistance-related reads per kilobase per genome equivalent (RPKG).

### Microbial host inference of RGs

The microbial origin of RGs was traced by assigning taxonomy to assembled contigs harboring target genes. Following alignment, the aligned reads harboring RGs were extracted and further used to link back to contigs (54). The resulting resistance-containing contigs were assigned a taxonomy via kaiju (55).

### Transferrable RGs and LGT in the fecal microbiome of dairy cattle

For acquired RG profiling, bwa-mem2 was used to align metagenomic sequences to ResFinder (v 4.1) (56). The resultant sequences were normalized in the same pipeline with MEGARes 2.0 analysis. WAAFLE (http://huttenhower.sph.harvard.edu/waafle) was utilized to predict LGT events in fecal microbiomes of cattle, and the frequency of LGT was normalized per 1K assembled genes before any downstream comparisons. The networks of LGT were constructed by the predicted edge matrixes and were visualized using the R package igraph (v 1.2.11) (57).

### Statistical analyses

Power analysis calculated before the experiment started determined a minimum sample size of 4 cattle. Each group was to have a power of at least 80% given the effect size of 0.3 with a type I error of 5% using G*Power 3.1.9.6 (58) based on the F-test of repeated measures within-between interaction ANOVA.

All the statistical analyses were performed using R (v 4.1.0). A mixed linear model (LMM) with post hoc tests in the R package lmerTest (59) was used to examine variances in alpha diversity for both microbiome and resistome results between the CON and HG groups. β-diversity was generated via Bray–Curtis distance, and the compositional variances across animals were assessed using an Adonis test in the R vegan package (60) with 9,999 permutations and then visualized using an NMDS plot. ANCOM-BC (61) was specifically used to perform microbial differential abundance analysis. An LMM was also employed to examine differences in the abundance of RGs between groups, with diet as the fixed effect and time (i.e., day) as the random variable. Differences were considered significant when *P* < 0.05.

## Data Availability

The fecal metagenome sequences were deposited into the NCBI Sequence Read Archive (SRA) under the accession number PRJNA970480.

## SUPPLEMENTAL MATERIAL

Supplemental material msystems.00810-23-s0001.docx Supplemental figures and table.

ASM does not own the copyrights to Supplemental Material that may be linked to, or accessed through, an article. The authors have granted ASM a non-exclusive, world-wide license to publish the Supplemental Material files. Please contact the corresponding author directly for reuse.
