# Supplementary material for: Fecal microbial gene transfer contributes to the high-grain diet-induced augmentation of aminoglycoside resistance in dairy cattle
Source: mSystems. 2023 Dec 12;9(1):e00810-23. doi: 10.1128/msystems.00810-23 (PMC10805029; doi:10.1128/msystems.00810-23)
Supplement: Supplemental material — Supplemental figures and table. [file msystems.00810-23-s0001.docx]

**Supplemental figure**


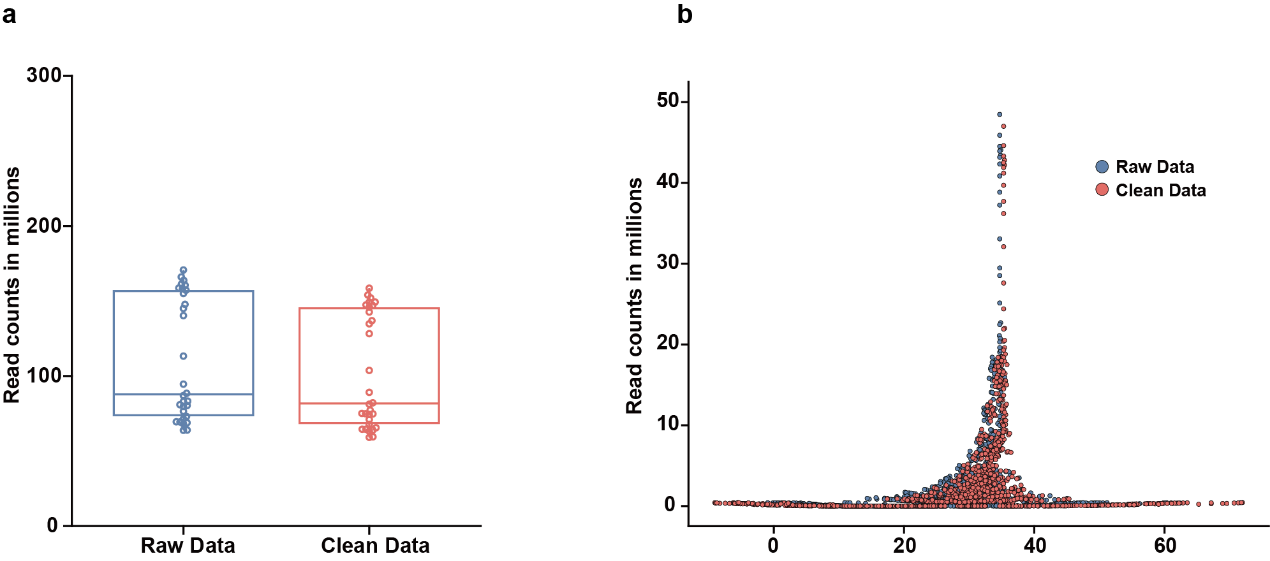


**Fig S1.** Quality control of metagenomic sequencing data. (**a**) The read counts per million for both raw data and clean data. (**b**) Quality score statistics of metagenomic sequences.


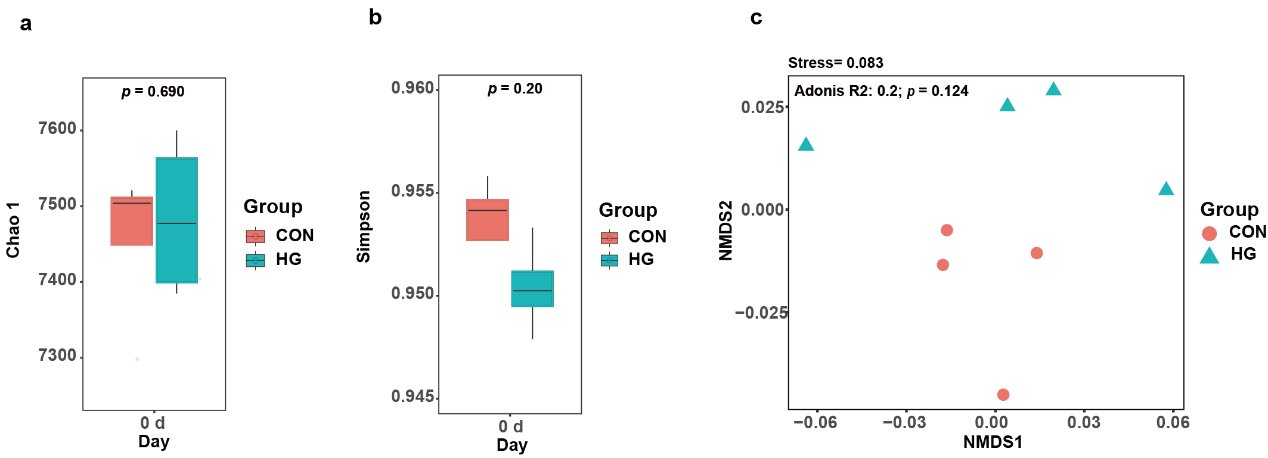
**Fig S2.** Fecal microbiome comparison in animals before dietary transition. **(a-b)** Alpha diversity (Chao1 and Simpson index) showed no difference in the fecal microbial community between CON and HG groups before dietary transition (Wilcoxon test, *P* > 0.05). **(c)** Non-metric multidimensional scaling (NMDS) analysis shows a similarity in fecal microbiota structure based on Bray-Curtis distance (Adonis, *P* > 0.05). CON: conventional diet; HG: high-grain diet


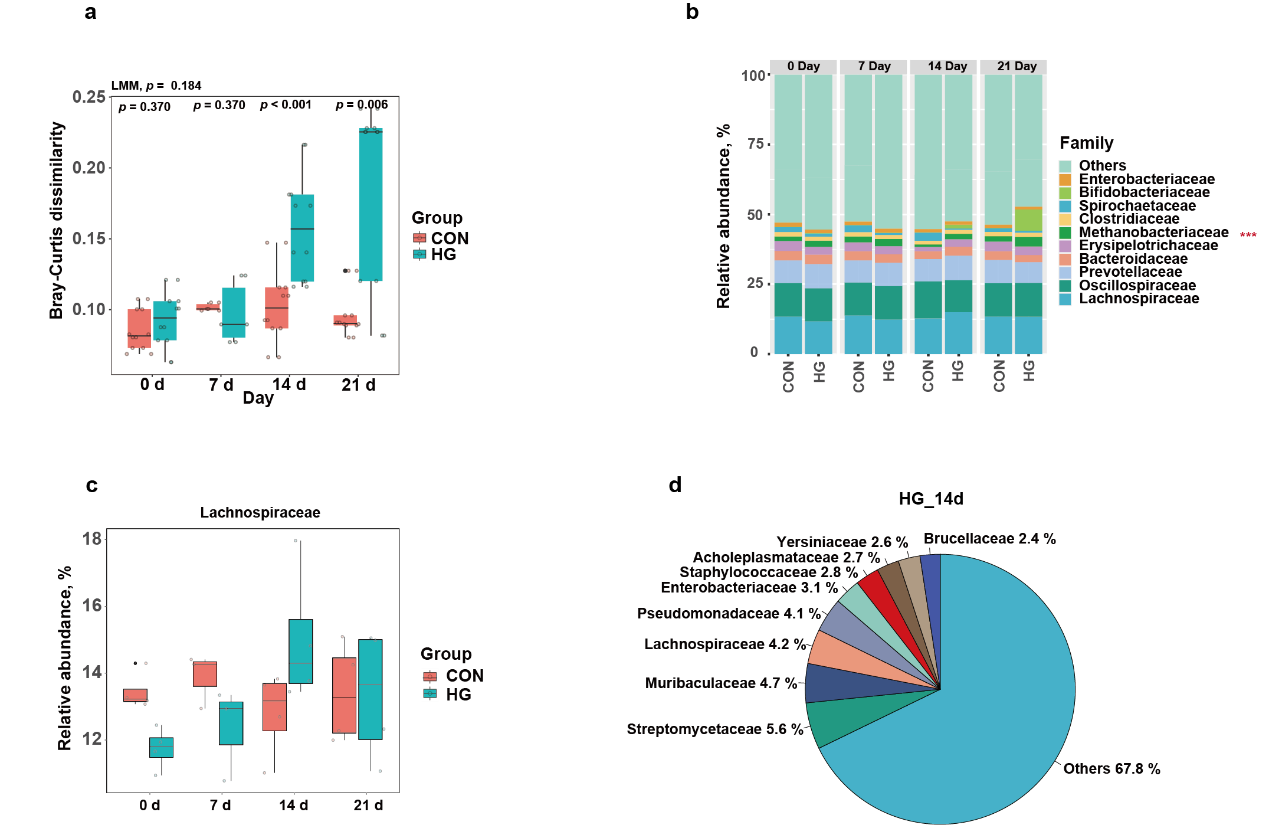
**Fig S3.** Alpha diversity and microbial composition of the fecal microbiome in dairy cattle. (**a**) Beta diversity of fecal microbiota across the CON and HG groups. (**b**) Relative abundance of the dominant family (> 1% in all samples) in both CON and HG groups. (ANCOM-BC, ***: *P* < 0.001) (**c**) Specific microbial composition of HG group at day 14 after dietary transition. (**d**) The relative abundance of Lachnospiraceae peaks at day 14 by high-grain dietary transition.


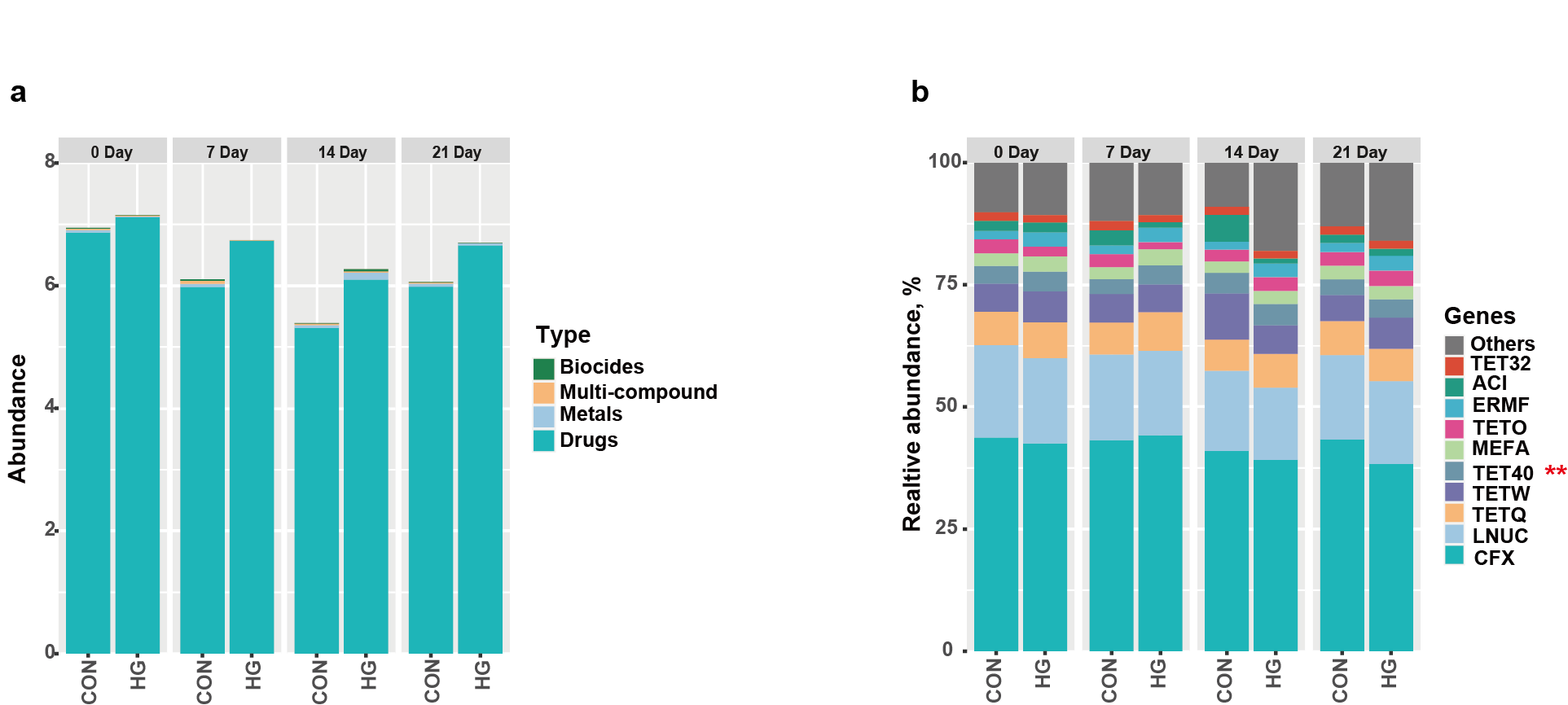
**Fig S4.** The composition of fecal resistome between the CON and HG groups. (**a**) The abundance of drug-resistance genes is greatest in cattle feces. (**b**) Dominant fecal resistome composition (> 1% in all samples) summarized at the gene level, showing the enrichment of *tet40* in the HG group (LMM, **: *P* < 0.05).

**Supplemental table**

**Table S1.** Ingredient and chemical composition of the conventional diet (CON) and high-grain diet (HG) (1).

| Item | CON | HG |
| --- | --- | --- |
| Ingredient, % of DM | | |
| Corn grain | 19.40 | 24.92 |
| Soybean | 13.50 | 13.48 |
| Barley | — | 12.00 |
| DDGS | 3.80 | 5.91 |
| CaCO_3_ | 0.80 | 1.48 |
| Ca(HCO_3_)_2_ | 1.10 | 0.92 |
| NaCl | 0.50 | 0.37 |
| Premix^1^ | 1.00 | 0.92 |
| Corn silage | 12.00 | 6.00 |
| American alfalfa hay | 24.00 | 17.00 |
| Australian oaten hay | 24.00 | 17.00 |
| Nutrient composition | | |
| DM, % | 46.77 | 48.03 |
| CP, % of DM | 16.16 | 16.12 |
| CF, % of DM | 3.05 | 3.05 |
| NDF, % of DM | 36.14 | 29.92 |
| NFC, ^2^ % of DM | 38.68 | 46.04 |
| Strach, % of DM | 17.96 | 27.82 |
| Ash, % of DM | 5.97 | 4.87 |
| Ca, % of DM | 1.14 | 1.18 |
| P, % of DM | 0.52 | 0.51 |
| NE_L_,^3^ % of DM | 1.57 | 1.64 |
| NFC/NDF | 0.93 | 1.54 |

DDGS: Dried distillers grains with solubles; DM: Dry matter; CP: crude protein; CF: Crude fat; NDF: neutral detergent fiber; NFC: non-fiber carbohydrate; NE: Net energy

^1^ Premix contained the following ingredients per kilogram of diet: vitamin A, 22.5 KIU/kg; vitamin D3, 5.0 KIU/kg; vitamin E, 37.5 IU/kg; vitamin K3, 5.0 mg/kg; Mn, 63.5 mg/kg; Zn, 111.9 mg/kg; Cu, 25.6 mg/kg; and Fe, 159.3 mg/kg.

^2^ NFC = 100 – (NDF %+ CP % + ether extract % + ash %)

^3^Calculated based on Ministry of P. R. China recommendations (2).

**REFERENCES**

1. Mu YY, Qi WP, Zhang T, Zhang JY, Mei SJ, Mao SY. 2021. Changes in rumen fermentation and bacterial community in lactating dairy cows with subacute rumen acidosis following rumen content transplantation. Journal of Dairy Science 104:10780-10795.

2. MOA. 2004. Feeding Standard of Dairy Cattle (NY/T 34–2004). MOA, Beijing, China.
